# Coronary Venous Mapping and Ablation for Ventricular Tachycardia

**DOI:** 10.19102/icrm.2023.14035

**Published:** 2023-03-15

**Authors:** Leah A. John, Jeffrey R. Winterfield

**Affiliations:** ^1^Division of Cardiovascular Medicine, Department of Medicine, Brigham and Women’s Hospital, Harvard Medical School, Boston, MA, USA; ^2^Division of Cardiology, Medical University of South Carolina, Charleston, SC, USA

**Keywords:** Ablation, coronary venous mapping, ischemic cardiomyopathy, ventricular tachycardia

## Abstract

Coronary venous mapping and ablation can be an effective strategy in targeting ventricular arrhythmias that arise from intramural or epicardial sites of origin. We discuss the case of a patient with ischemic cardiomyopathy referred to our center for index ventricular tachycardia ablation after receiving multiple shocks from his implantable cardioverter-defibrillator who underwent coronary venous mapping and ablation as an adjunct to endocardial ventricular tachycardia ablation.

## Introduction

Coronary venous mapping and ablation can be an effective strategy in targeting ventricular arrhythmias that arise from intramural or epicardial sites of origin. We describe a case of coronary venous mapping and ablation as an adjunct to endocardial ventricular tachycardia (VT) ablation in a patient with ischemic cardiomyopathy.

## Case presentation

A 69-year-old man with a history of atrial fibrillation, coronary artery disease, and prior coronary artery bypass surgery was referred to our center for index VT ablation after receiving multiple shocks from his biventricular implantable cardioverter-defibrillator (Quadra Assura™; Abbott, Chicago, IL, USA). He was started on amiodarone in February 2021 and underwent left heart catheterization, which revealed 3 patent grafts and a single occluded selective saphenous vein graft to the right coronary artery.

The patient was brought to the electrophysiology laboratory, and general anesthesia was administered by an anesthesia provider. We began with programmed stimulation from the right ventricle (RV) and easily induced clinical VT 1 with double extra-stimuli. The morphology of VT 1 was left bundle branch block (LBBB), +II, −III, and +precordial leads with a cycle length (CL) of 382 ms. VT 1 was not hemodynamically tolerated, and pace-termination attempts switched the presentation from VT 1 to VT 2, which involved LBBB, a left superior axis, and rS precordial leads with a CL of 434 ms. We initially created a substrate map of the RV using the Advisor™ HD Grid catheter and EnSite Precision™ mapping system (both Abbott). The timing of the His was late relative to the QRS interval, and we focused on the left ventricle (LV). We advanced the EPstar catheter (Baylis Medical, Montreal, Quebec, Canada) into the middle cardiac vein (MCV) for coronary venous mapping.

We created an LV substrate map using a combination of retrograde and transseptal approaches. We noted a large infero-posterior aneurysm extending between the mitral valve annulus and the posteromedial papillary muscle. We also observed late potentials (LPs) and local abnormal ventricular activity and used latest late detection during RV pacing to identify areas of slow conduction according to the isochronal late activation mapping (ILAM) strategy.

**Figure 1:**
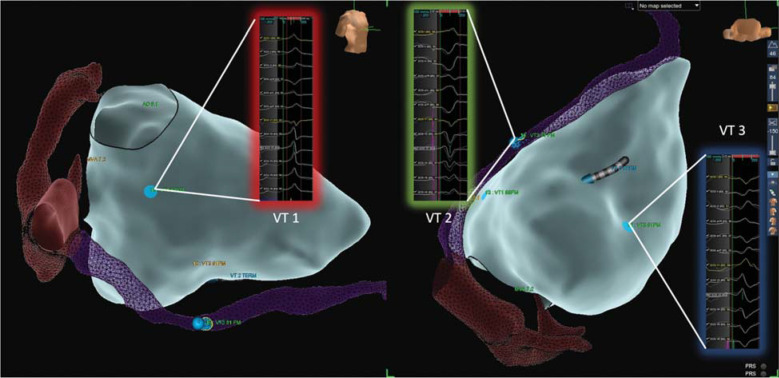
Ventricular tachycardia morphologies and sites of best pace-maps. *Abbreviation:* VT, ventricular tachycardia.

**Figure 2:**
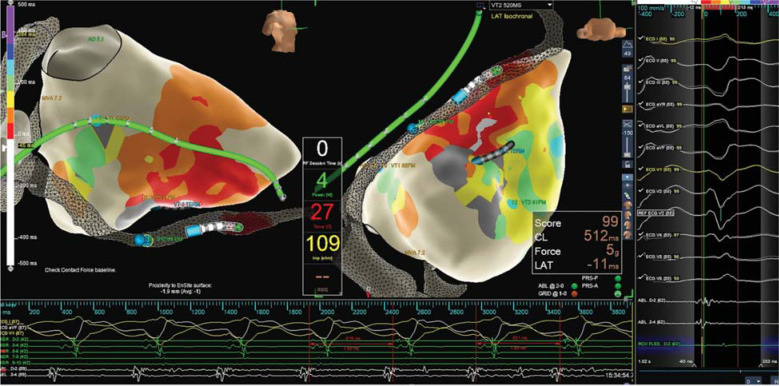
Slowing of ventricular tachycardia 2 with ablation from the TactiCath™ ablation catheter positioned within the middle cardiac vein. *Abbreviation:* VT, ventricular tachycardia.

With the Advisor™ HD Grid catheter positioned along the atrioventricular basal mid-septum and just anterior to the His-bundle region, we had 88% pace-maps to VT 1. We noted low voltage along the postero-inferior LV with associated LPs and fractionated signals. During LV substrate mapping, VT 3 was spontaneously induced, for which we had 91% pace-maps along the basal mid-inferior LV septum **(Figure 1)**. The VT 3 morphology was right bundle branch block with rightward, indeterminate, V4 transition and a CL of 431 ms. Using a combination of ILAM and pace-mapping approaches, we targeted zones of slow conduction and the best pace-maps for each of the VTs. We initially focused on the His region and mid-inferior LV septum, targeting sites previously tagged for ablation. We attempted re-induction post-ablation and re-induced VT 2. The best pace-maps for VT 2 were 86% within the MCV **(Figure 1)**. With entrainment here, we manifested fusion with a post-pacing interval tachycardia CL (PPI-TCL) of 30 ms, suggesting that the MCV was an outerloop site. We decided to exchange the EPstar catheter for the TactiCath™ F/J ablation catheter (Abbott) and positioned this catheter within the MCV. We delivered several radiofrequency (RF) lesions within the MCV and noted roughly 100-ms slowing of VT 2 during ablation **(Figure 2)**.

**Figure 3:**
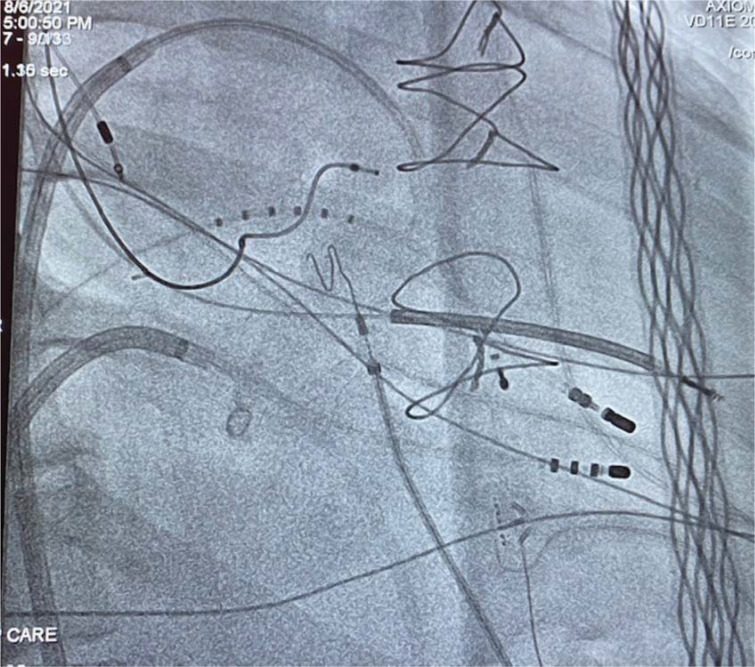
A FlexAbility ablation catheter positioned in the left ventricular endocardium just appositional to the TactiCath™ ablation catheter positioned within the middle cardiac vein.

**Figure 4:**
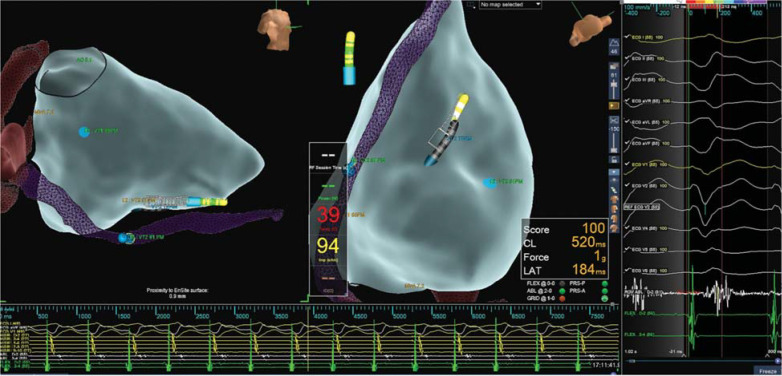
Ventricular tachycardia 2 termination with radiofrequency energy and corresponding signals on the FlexAbility and TactiCath™ ablation catheters.

Because we still did not have VT termination, we decided to use a second ablation catheter, FlexAbility F (Abbott), which was positioned within the LV just appositional to sites of ablation from the MCV **(Figure 3)**. At this point, we noted mid-diastolic potentials along the mid-inferior LV wall, and entrainment at this site was concealed with a PPI-TCL of 0 ms. At this point, we switched to half-normal saline and delivered RF energy here with successful termination of VT. We performed final induction and re-induced VT 2. With the TactiCath™ ablation catheter still positioned within the MCV, we delivered additional RF energy from the MCV (using half-normal saline with 20 W power with slow titration). Finally, using sequential RF delivery from the TactiCath™ (within the MCV) and FlexAbility catheters positioned appositionally within the LV endocardium, we had successful termination of VT 2 **(Figure 4)**.

## Discussion

Coronary venous mapping and ablation of VT can be an effective strategy to target VT, particularly in instances where a mid-myocardial or epicardial substrate may exist.^1^ Mapping and ablation within the coronary venous system (CVS) in conjunction with ablation and mapping at appositional endocardial sites can be successful in selected cases. Our case highlights the use of CVS mapping and ablation in combination with endocardial mapping and ablation plus entrainment maneuvers as effective strategies in targeting VT. With CVS mapping, it is important to appreciate the adjacent anatomical structures and consider potential complications with such an approach.

## References

[1] Karimianpour A, Badertscher P, Payne J, Field M, Gold MR, Winterfield JR Epicardial mapping and ablation of ventricular tachycardia from coronary venous system in post-coronary bypass patients [published online ahead of print 18 May 2022]. J Interv Card Electrophysiol.

